# Brain age gap, dementia risk factors and cognition in middle age

**DOI:** 10.1093/braincomms/fcae392

**Published:** 2024-11-19

**Authors:** James D Stefaniak, Elijah Mak, Li Su, Stephen F Carter, Maria-Eleni Dounavi, Graciela Muniz Terrera, Katie Bridgeman, Karen Ritchie, Brian Lawlor, Lorina Naci, Ivan Koychev, Paresh Malhotra, Craig W Ritchie, John T O’Brien

**Affiliations:** Department of Psychiatry, University of Cambridge School of Clinical Medicine, Cambridge CB2 0SP, UK; Cambridgeshire and Peterborough NHS Foundation Trust, Cambridge CB21 5EF, UK; Department of Psychiatry, University of Cambridge School of Clinical Medicine, Cambridge CB2 0SP, UK; Department of Psychiatry, University of Cambridge School of Clinical Medicine, Cambridge CB2 0SP, UK; Department of Neuroscience, University of Sheffield, Sheffield S10 2TN, UK; Department of Psychiatry, University of Cambridge School of Clinical Medicine, Cambridge CB2 0SP, UK; Department of Psychiatry, University of Cambridge School of Clinical Medicine, Cambridge CB2 0SP, UK; Edinburgh Dementia Prevention, Centre for Clinical Brain Sciences, University of Edinburgh, Edinburgh EH4 2XU, UK; Department of Social Medicine, Ohio University, Athens OH 45701, USA; Edinburgh Dementia Prevention, Centre for Clinical Brain Sciences, University of Edinburgh, Edinburgh EH4 2XU, UK; INSERM, INM, U1061 Neuropsychiatrie, Montpellier, France; Global Brain Health Institute, Trinity College Dublin, University of Dublin, Dublin 2, D02 X9W9, Ireland; Global Brain Health Institute, Trinity College Dublin, University of Dublin, Dublin 2, D02 X9W9, Ireland; Department of Psychiatry, Oxford University, Oxford OX3 7JX, UK; Department of Brain Sciences, Imperial College London, London W12 0NN, UK; Edinburgh Dementia Prevention, Centre for Clinical Brain Sciences, University of Edinburgh, Edinburgh EH4 2XU, UK; Scottish Brain Sciences, Edinburgh EH12 9DQ, UK; Department of Psychiatry, University of Cambridge School of Clinical Medicine, Cambridge CB2 0SP, UK; Cambridgeshire and Peterborough NHS Foundation Trust, Cambridge CB21 5EF, UK

**Keywords:** dementia, brain age gap, middle age, cognition, machine learning

## Abstract

Brain Age Gap has been associated with dementia in old age. Less is known relating brain age gap to dementia risk-factors or cognitive performance in middle-age. Cognitively healthy, middle-aged subjects from PREVENT-Dementia had comprehensive neuropsychological, neuroimaging and genetic assessments. Brain Ages were predicted from T1-weighted 3T MRI scans. Cognition was assessed using the COGNITO computerized test battery. 552 middle-aged participants (median [interquartile range] age 52.8 [8.7] years, 60.0% female) had baseline data, of whom 95 had amyloid PET data. Brain age gap in middle-age was associated with hypertension (*P* = 0.007) and alcohol intake (*P* = 0.008) but not apolipoprotein E epsilon 4 allele (*P* = 0.14), amyloid centiloids (*P* = 0.39) or cognitive performance (*P* = 0.74). Brain age gap in middle-age is associated with modifiable dementia risk-factors, but not with genetic risk for Alzheimer's disease, amyloid deposition or cognitive performance. These results are important for understanding brain-age in middle-aged populations, which might be optimally targeted by future dementia-preventing therapies.

## Teaser

Stefaniak *et al*. report that brain age gap in middle-age is associated with modifiable dementia risk-factors (hypertension and alcohol intake), but not with Alzheimer’s disease-specific risk-factors (*APOE4* or amyloid deposition), nor cognitive performance. Lifestyle risk-factor modification needs to start at, or before, midlife to optimize brain health.

## Graphical Abstract

**Graphical Abstract:**
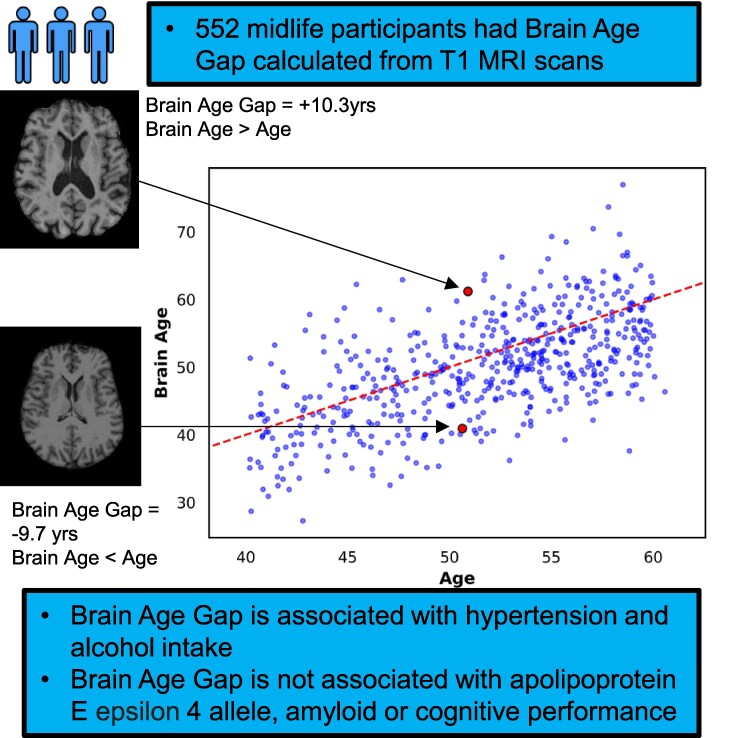

## Introduction

Brain age gap (BAG) represents the difference between an individual’s predicted age, derived from machine learning models trained on magnetic resonance imaging (MRI) data, and their chronological age.^1^ BAG is increased in mild cognitive impairment (MCI) and dementia.^2^ In MCI, elevated BAG is associated with a greater risk of conversion to dementia^3,4^ and with poorer cognitive performance.^4^ In asymptomatic older people, BAG has been associated with cardiovascular risk-factors,^5^ alcohol consumption,^6^ cognitive performance,^7^ amyloid deposition^8^ and an increased risk of subsequently developing dementia.^9^

While BAG has been extensively studied in clinical samples and healthy older people, little has been published relating BAG to dementia risk or cognitive performance in middle-age. One study reported an association between BAG and IQ in a cohort of individuals all aged 45 years.^10^ However, other studies have failed to find an association between BAG and cognition in healthy older people.^4^ It is important to clarify the relationship between BAG, dementia risk-factors and cognition in midlife in order to inform its putative role as a preclinical biomarker. We therefore investigated BAG in the PREVENT-Dementia cohort of middle-aged individuals enriched for a family history of dementia.

This analysis was pre-registered and adequately powered to find an association between BAG and cognitive performance in middle age. However, statistical associations do not necessarily imply predictive value,^11^ and it is possible that any relationship between BAG and cognitive performance is non-linear and not adequately captured by standard linear models. This study therefore performed an additional set of predictive modelling analyses in which a variety of linear and non-linear machine learning algorithms tested whether BAG and cognitive performance can predict each other.

## Materials and methods

### Setting and participants

Full details of the PREVENT-Dementia programme are described elsewhere.^12^ Participants were cognitively normal, middle-aged (40–59 years) subjects recruited from multiple sources across five sites with a target of 50% participants having a history of parental dementia. The research was approved by the London–Camberwell St Giles NHS ethics committee. All subjects provided written informed consent according to the declaration of Helsinki. 702 participants were recruited from five study sites (West London (210), Edinburgh (224), Cambridge (100), Oxford (68) and Dublin (100)). After excluding participants without imaging (*n* = 56), incidental MRI findings (*n* = 11) or missing risk-factor (*n* = 74) or cognitive (*n* = 9) data, 552 participants were included. Ninety-five participants had florbetaben amyloid PET/CT imaging data.

### Risk-factors and cognition

Midlife risk-factors for dementia, established from previous research,^13^ included: hypertension, obesity or hearing disorder; alcohol intake (number of glasses of wine/beer per week), head trauma (lifetime number of head blows) and possession of the apolipoprotein E epsilon 4 (*APOE4*) allele (genotyping used QuantStudio12K-Flex).

Cognition was assessed using 17 variables from the COGNITO computerized test battery.^14^ Subsets of these variables represented: attention (visual attention, auditory attention, visual and auditory attention); language (sentence comprehension, verbal fluency and vocabulary test); memory (immediate recall, delayed recall, face recall and name-face association; narrative recall; implicit memory); and visuospatial function (form matching, visuospatial span, logical series, visuospatial construction and Stroop test). Principal component analysis (PCA) was used to reduce the number of variables for analysis. The first unrotated principal component (PC) was used to represent each aspect of cognition in statistical analyses (‘Cognition PC’ using PCA restricted to one component on all 17 variables or ‘Attention PC’, ‘Language PC’, ‘Memory PC’ and ‘Visuospatial PC’ using their respective variables).

### BAG estimation

All included subjects had a T1-weighted magnetisation-prepared rapid gradient echo MRI scan with the following acquisition parameters: repetition time = 2.3 s, echo time = 2.98 ms, 160 slices, flip angle = 9° and voxel size = 1mm^3^ isotropic. All MRI scans were acquired on a 3T Siemens scanner with the following models: Verio (West London, Edinburgh); Prisma (Oxford, Edinburgh); Prisma fit (Cambridge); and Skyra (Dublin, Edinburgh).

Brain age was estimated from raw T1-weighted MRI scans using a publicly accessible model that had been developed using Gaussian process regression by an external party.^15^ Input to the model consists of raw T1-weighted MRI scans that have been segmented into white and grey matter and normalized using SPM12. The model was reported to have performed excellently in an independent validation dataset of 611 individuals aged 18–90 years (*r* = 0.95 between chronological and predicted age).^15,16^ None of the participants in the current study had been used to train the model. BAG was calculated as chronological age subtracted from brain age.

### Pre-registration and power analysis

The analysis plan was pre-registered (https://aspredicted.org/M88_D9H). Based on the standardized effect size of 0.2 from a previous paper,^10^ an *a priori* power analysis suggested that 343 participants were needed for 80% power to detect an association between BAG and cognition in middle age. A retrospective power calculation suggests our sample size of 552 had 96% power.

### Statistical analysis

Statistical significance was defined as *P* < 0.05 and two-sided. Bonferroni correction was applied to the significance threshold whenever multiple unifactorial tests were performed on the same dataset (reported *P*-values are uncorrected for transparency). Multiple linear robust regression models tested hypotheses that: BAG is positively associated with dementia risk-factors; and increased BAG is associated with worse cognition. Linear models controlled for sex, scanning site, years of education, age, age-squared^15^ and age*sex.^17^

Analyses were performed using python v3.8.16, with data handling using pandas v1.3.5 and numpy v1.21.6 and statistical analyses using statsmodels v0.12.2 and sklearn v1.0.2. Robust linear regression models were implemented using ‘statsmodels’ with default settings (Huber’s T norm and median absolute deviation scaling).

### Predictive modelling

Statistical associations do not necessarily imply predictive value.^11^ Furthermore, it is possible that any relationship between BAG and cognition is non-linear (rather than linear). Thus, a range of both linear and non-linear machine learning (ML) regression algorithms were used to examine the predictive relationship between BAG and cognition. Linear regression algorithms included ordinary least squares, ridge, and least absolute shrinkage and selection operator, while non-linear regression algorithms included nu-support vector and extreme gradient boosting. Models were trained to predict BAG from cognition (the 17 raw COGNITO variables), and conversely, to predict cognitive performance (cognition PCs) from demographic/risk-factor data, with and without BAG included. Random-permutation nested cross-validation with hyperparameter optimisation was employed to provide unbiased estimates of model performance, using mean absolute deviation (MAD) as the metric of the mean generalisation error. Bootstrapping (1000 iterations) was used to obtain 95% confidence intervals (CIs) for the MAD estimates.

### *Post hoc* correction of brain age for site and age

Additional *post hoc* analyses were performed to remove residual scanning-site and age effects on the predicted Brain Ages.

To remove hypothetical scanning site effects, we employed the ComBat method for data harmonisation without the empirical Bayes function, in line with our recent studies.^18^ The default empirical Bayes option in ComBat estimates parameters for the scaling and shifting of features based on all features in the dataset. Since only a single feature (brain age) was being harmonized, estimating distributional parameters from a single feature avoids potential issues with single-feature distributional parameter estimation and overfitting.

To remove residual age-bias effects, the ComBat site harmonized brain ages from the previous step were then age-bias corrected according to a previously published method.^19^ The dataset containing all included participants was split into 10 ‘folds’. For each fold, a linear regression model was trained to predict brain age from chronological age using the nine remaining folds as a ‘training dataset’, with the formula: brain age = alpha × chronological age + beta. The derived values of alpha and beta from each fold’s training dataset were then used to correct predicted brain age in the left-out fold with: age-bias corrected brain age = brain age + [chronological age—(alpha × chronological age + beta)]. This was repeated for each of the 10-folds in turn.

‘Age and Site Corrected Brain Age Gap’ (‘ASC-BAG’) was calculated as chronological age subtracted from Age-Bias Corrected, ComBat Site Harmonized Brain Age.

### *Post hoc* analyses

All association analyses using BAG in the present study were then repeated using ‘ASC-BAG’, to confirm that the observed results were not due to scanning-site and age effects on the predicted brain ages.

*Post hoc* linear models with ‘ASC-BAG’ as the dependent variable, controlled for sex and years of education but not age, age-squared,^20^ age*sex^17^ nor scanning-site. *Post hoc* linear models used *APOE4* homozygosity (homozygous versus not homozygous) instead of *APOE4* carrier status, after reviewer comments.

We did not repeat predictive modelling analyses using ‘ASC-BAG’ instead of BAG, because performing ComBat site harmonisation and then age-bias correction on the predicted brain ages caused information to ‘leak’ between training and testing datasets during subsequent predictive modelling analyses, which would have positively biased predictive performance of models on held-out testing datasets.

See the online supplement for additional methodological details.

## Results

**Figure 1:**
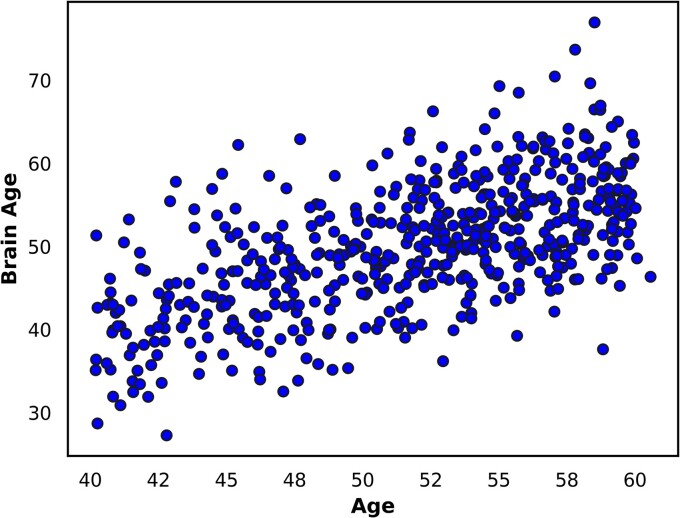
**Scatter plot of brain age versus chronological age.** Scatter plot showing the relationship between brain age and chronological age for the 552 included participants. Brain age correlated with chronological age (Spearman’s rho = 0.60, *P* = 4.7 × 10^−55^).

At the group level, this cohort’s brain ages were younger than their chronological ages (median 52.8 years), yielding a median (interquartile range) BAG of −2.0 (7.8) years (Supplementary Table S1). BAG varied between individuals, ranging from −21.2 years to +18.4 years. The median (interquartile range) ‘ASC-BAG’ was −0.2 (8.1) years (Supplementary Table S2). Brain Age correlated with chronological age (Spearman’s rho = 0.60, *P* = 4.7 × 10^−55^) (Fig. 1), in keeping with previous literature.^21^ Age was negatively associated with BAG (Spearman’s rho = −0.13, *P* = 0.003), and there were significant differences in BAG between scanning sites (Kruskall–Wallis H test, χ^2^ = 22.96, *P* = 0.0001) (Supplementary Table S1). As expected, age was no longer associated with ‘ASC-BAG’ (Spearman’s rho = −0.01, *P* = 0.86), and there were not significant differences in ‘ASC-BAG’ between scanning sites (Kruskall–Wallis H test, χ2 = 0.06, *P* = 0.99) (Supplementary Table S2).

### BAG and dementia risk-factors

A multiple linear robust regression model including modifiable dementia risk-factors identified hypertension (B = 2.31, *P* = 0.007) and alcohol intake (B = 0.11, *P* = 0.008) as being significantly associated with increased BAG (Table 1). A *post hoc* model including modifiable dementia risk-factors identified hypertension (B = 2.23, *P* = 0.01) and alcohol intake (B = 0.10, *P* = 0.01) as being significantly associated with increased ‘ASC-BAG’ (Supplementary Table S3).

**Table 1:** Multiple linear regression model of participant characteristics and modifiable dementia risk-factors with BAG

Participant characteristic	Multiple linear regression with BAG	
	B (95% CI)	*P* value
Intercept	−24.50 (−69.12 to 20.12)	0.28
Medical history of hypertension	2.31 (0.62 to 3.99)	**0**.**007**
Medical history of obesity	0.24 (−1.31 to 1.79)	0.76
Medical history of hearing disorder	0.33 (−1.19 to 1.86)	0.67
Sex—male	−4.36 (−14.04 to 5.33)	0.38
Scanning site (compared to site A):		
Site B	1.20 (−0.07 to 2.48)	0.07
Site C	−2.62 (−4.24 to −1.01)	**0.001**
Site D	−1.14 (−2.92 to 0.64)	0.21
Site E	0.77 (−0.94 to 2.48)	0.38
Alcohol	0.11 (0.03 to 0.19)	**0**.**008**
Head trauma	0.02 (−0.09 to 0.12)	0.76
Age	1.21 (−0.56 to 2.98)	0.18
Age*sex	−0.08 (−0.26 to 0.11)	0.41
Years of education	−0.08 (−0.24 to 0.08)	0.32
Age^2^	−0.01 (−0.03 to 0.004)	0.14

Robust multiple linear regression of participant characteristics and modifiable dementia risk factors against BAG. This analysis was performed on the 552 included participants. Statistically significant *P*-values are highlighted in bold.

Abbreviations: B, unstandardized regression coefficient; CI, confidence interval.

A model investigating the genetic risk of Alzheimer’s disease did not find an association between *APOE4* carrier status and BAG (B = 0.79, *P* = 0.14) (Supplementary Table S4). A *post hoc* model did not find an association between *APOE4* homozygous status and ‘ASC-BAG’ (B = 0.48, *P* = 0.68) (Supplementary Table S5).

A third model did not find an association between amyloid centiloids^22^ and BAG (B = −0.03, *P* = 0.39) (Supplementary Table S6). A *post hoc* model did not find an association between amyloid centiloids and ‘ASC-BAG’ (B = −0.04, *P* = 0.32) (Supplementary Table S7).

### BAG and cognition

Virtually all COGNITO variables loaded negatively on ‘Cognition PC’, ‘Attention PC’, ‘Language PC’, ‘Memory PC’ and ‘Visuospatial PC’ (Supplementary Table S8), meaning that lower PC scores indicated better cognitive performance.

BAG was not significantly associated with ‘Cognition PC’ (B = 0.004, *P* = 0.74) (Table 2), ‘Attention PC’ (B = 0.005, *P* = 0.25) (Supplementary Table S9), ‘Language PC’ (B = 0.01, *P* = 0.09) (Supplementary Table S10), ‘Memory PC’ (B = −0.01, *P* = 0.61) (Supplementary Table S11) or ‘Visuospatial PC’ (B = 0.01, *P* = 0.18) (Supplementary Table S12). Similarly, ‘ASC-BAG’ was not significantly associated with ‘Cognition PC’ (B = 0.004, *P* = 0.72) (Supplementary Table S13), ‘Attention PC’ (B = 0.004, *P* = 0.28) (Supplementary Table S14), ‘Language PC’ (B = 0.01, *P* = 0.09) (Supplementary Table S15), ‘Memory PC’ (B = −0.01, *P* = 0.65) (Supplementary Table S16) or ‘Visuospatial PC’ (B = 0.01, *P* = 0.20) (Supplementary Table S17). More years of education was associated with better cognitive performance on ‘Cognition PC’ (B = −0.14, *P* = 4.7 × 10^−11^) (Table 2), ‘Language PC’ (B = −0.13, *P* = 1.4 × 10^−20^) (Supplementary Table S10), ‘Memory PC’ (B = −0.06, *P* = 0.003) (Supplementary Table S11) and ‘Visuospatial PC’ (B = −0.07, *P* = 1.2 × 10^−5^) (Supplementary Table S12).

**Table 2:** Multiple linear regression model of participant characteristics and BAG with ‘cognition PC’

Participant characteristic	Multiple linear regression with ‘cognition PC’	
	*B* (95% CI)	*P* value
Intercept	11.90 (−0.05 to 23.86)	0.05
Sex—male	−0.84 (−3.44 to 1.76)	0.53
Scanning site (compared to site A):		
Site B	−0.20 (−0.54 to 0.14)	0.25
Site C	0.37 (−0.07 to 0.80)	0.10
Site D	−0.03 (−0.51 to 0.45)	0.91
Site E	0.69 (0.24 to 1.14)	**0.003**
BAG	0.004 (−0.02 to 0.03)	0.74
Age	−0.37 (−0.85 to 0.10)	0.13
Age*sex	−0.03 (−0.08 to 0.02)	0.26
Years of education	−0.14 (−0.19 to −0.10)	**4.7 × 10^−11^**
Age^2^	0.004 (−0.001 to 0.009)	0.09

Robust multiple linear regression of participant characteristics and BAG against ‘Cognition PC’. This analysis was performed on the 552 included participants. Statistically significant *P*-values are highlighted in bold.

Abbreviations: B, unstandardized regression coefficient; CI, confidence interval; PC, principal component.

### Predictive modelling

COGNITO data could not be used to predict BAG more accurately than a mean-predicting ‘Dummy’ baseline (Table 3). This was shown by overlap between the 95% CIs of mean MAD for all regression algorithms and that of a mean-predicting ‘Dummy’ algorithm (Table 3). Similarly, BAG did not significantly improve the ability of any regression algorithm to predict any of the cognition PCs when added to demographic/risk-factor data (Supplementary Tables S18–S22). This was shown by overlap between the 95% CIs of mean MAD when demographic/risk-factor data were the independent variables and when demographic/risk-factor plus BAG were the independent variables (Supplementary Tables S18–S22). Furthermore, non-linear regression algorithms were not significantly superior to linear regression algorithms at explaining the relationship between BAG and cognition (Table 3 and Supplementary Tables S18–S22). This was shown by overlap between the 95% CIs of mean MAD of linear (ordinary least squares, ridge, least absolute shrinkage and selection operator) and non-linear (nu-Support Vector, XGBoost) regression algorithms (Table 3 and Supplementary Tables S18–S22).

**Table 3:** Multivariable regression models predicting BAG from cognitive data

Dependent variable	Independent variables	Regression algorithm	Mean MAD	95% CI of mean MAD
BAG	COGNITO	Dummy	4.99	4.37 to 5.32
	COGNITO	OLS	4.81	4.46 to 5.44
	COGNITO	Ridge	4.85	4.41 to 5.34
	COGNITO	LASSO	5.00	4.43 to 5.37
	COGNITO	NuSVR	4.81	4.47 to 5.45
	COGNITO	XGBoost	4.78	4.94 to 5.89

Multivariable regression models predicting BAG from cognitive data. COGNITO represents the 17 COGNITO variables used to assess cognition. This analysis was performed on the 552 included participants.

Abbreviations: CI, confidence interval; LASSO, least absolute shrinkage and selection operator; MAD, mean absolute deviation; NuSVR, nu-support vector regression; OLS, ordinary least squares; XGBoost, extreme gradient boosting.

## Discussion

This cohort had a negative median BAG, meaning their Brain Ages were younger than their chronological ages, on average. This might indicate ‘healthier volunteer’ selection bias.^23^ Nevertheless, individual BAGs varied widely, indicating that even in middle-age, substantial inter-individual heterogeneity exists.

BAG in middle-age was associated with hypertension and alcohol intake, which is known midlife risk-factors associated with later-life dementia.^13^ This suggests that BAG might be modifiable by taking action to address such lifestyle risk factors. It also indicates that BAG in middle age might prove to be associated with subsequent risk of dementia in future longitudinal studies.

BAG was not associated with genetic risk of Alzheimer’s disease (*APOE4*) or amyloid deposition in the present study. Previous studies in asymptomatic elderly individuals have found associations between BAG and amyloid deposition and *APOE4* status,^8^ although an association between BAG and *APOE4* status has not been found in other work.^16^ The present study’s lack of association between Alzheimer's disease-specific risk factors (*APOE4*, amyloid deposition) and BAG, but the positive association between modifiable lifestyle risk factors and BAG, suggests that BAG in middle-age might be more related to risk factors for dementia and poor brain health ‘in general’ rather than being specifically associated with Alzheimer's disease-specific risk factors in particular.^24^ The association also suggests that lifestyle risk factor modification needs to start at, or before, mid-life in order to optimize brain health.

The lack of association between BAG and education suggests that the protective effect of education against late-life dementia^13^ is mediated through mechanisms that operate independently of brain age in midlife.

A remarkably consistent finding from this pre-registered, well-powered study was the complete lack of any association between BAG in middle-age and cognitive performance. This was despite there being a plausible and expected association between more education and better cognitive performance, which suggested our measures of cognitive performance were valid. Furthermore, a wide range of both linear and non-linear ML algorithms found no predictive value between BAG in middle-age and cognitive performance. Taken together, these results suggest that BAG is not related to cognitive performance in middle-age. This is important for better understanding brain age in middle-age populations who might be the target of future dementia-preventing therapies. It also suggests that BAG might provide information, in addition to cognitive testing, when assessing mid-life dementia risk.

This study has limitations, including the lack of data about incident dementia and the lower sample size with PET. ComBat site harmonisation of brain age was performed after calculating brain age (rather than performing correction before computing brain age). The subjects included in this study were mainly Caucasian (Supplementary Table S2); future research might investigate whether the BAG results reported herein generalize to other racial groups. Longitudinal follow-up of subjects by the PREVENT-dementia study should seek to clarify whether BAG in midlife is associated with subsequent risk of developing all-cause or Alzheimer’s dementia. Future work should investigate whether midlife risk-factor modification can improve midlife BAG and subsequent dementia risk.

## Supplementary Material

fcae392_Supplementary_Data

## Data Availability

The data that support the findings of this study might be available on request from the corresponding author. The code used in this work is available at https://github.com/js762/BAG.

## References

[1] Franke K, Gaser C. Ten years of BrainAGE as a neuroimaging biomarker of brain aging: What insights have we gained? Front Neurol. 2019;10:789. 31474922 10.3389/fneur.2019.00789 PMC6702897

[2] Kaufmann T, van der Meer D, Doan NT, et al Common brain disorders are associated with heritable patterns of apparent aging of the brain. Nat Neurosci. 2019;22(10):1617–1623. 31551603 10.1038/s41593-019-0471-7 PMC6823048

[3] Gaser C, Franke K, Klöppel S, Koutsouleris N, Sauer H. Alzheimer’s disease neuroimaging initiative. BrainAGE in mild cognitive impaired patients: Predicting the conversion to Alzheimer’s disease. PLoS One. 2013;8(6):e67346. 23826273 10.1371/journal.pone.0067346 PMC3695013

[4] Löwe LC, Gaser C, Franke K. Alzheimer’s disease neuroimaging initiative. The effect of the APOE genotype on individual BrainAGE in normal aging, mild cognitive impairment, and Alzheimer’s disease. PLoS One. 2016;11(7):e0157514. 27410431 10.1371/journal.pone.0157514 PMC4943637

[5] de Lange AMG, Anatürk M, Suri S, et al Multimodal brain-age prediction and cardiovascular risk: The whitehall II MRI sub-study. Neuroimage. 2020;222:117292. 32835819 10.1016/j.neuroimage.2020.117292 PMC8121758

[6] Franke K, Gaser C, Manor B, Novak V. Advanced BrainAGE in older adults with type 2 diabetes mellitus. Front Aging Neurosci. 2013;5:90. 24381557 10.3389/fnagi.2013.00090 PMC3865444

[7] Liem F, Varoquaux G, Kynast J, et al Predicting brain-age from multimodal imaging data captures cognitive impairment. Neuroimage. 2017;148:179–188. 27890805 10.1016/j.neuroimage.2016.11.005

[8] Cumplido-Mayoral I, Garcia-Prat M, Operto G, et al Biological brain age prediction using machine learning on structural neuroimaging data: Multi-cohort validation against biomarkers of Alzheimer’s disease and neurodegeneration stratified by sex. Elife. 2023;12:e81067. 37067031 10.7554/eLife.81067 PMC10181824

[9] Wang J, Knol MJ, Tiulpin A, et al Gray matter age prediction as a biomarker for risk of dementia. Proc Natl Acad Sci U S A. 2019;116(42):21213–21218. 31575746 10.1073/pnas.1902376116 PMC6800321

[10] Elliott ML, Belsky DW, Knodt AR, et al Brain-age in midlife is associated with accelerated biological aging and cognitive decline in a longitudinal birth cohort. Mol Psychiatry. 2021;26(8):3829–3838. 31822815 10.1038/s41380-019-0626-7 PMC7282987

[11] Poldrack RA, Huckins G, Varoquaux G. Establishment of best practices for evidence for prediction: A review. JAMA Psychiatry. 2020;77(5):534–540. 31774490 10.1001/jamapsychiatry.2019.3671 PMC7250718

[12] Ritchie CW, Ritchie K. The PREVENT study: A prospective cohort study to identify mid-life biomarkers of late-onset Alzheimer’s disease. BMJ Open. 2012;2(6):e001893. 10.1136/bmjopen-2012-001893 PMC3533047 23166135

[13] Livingston G, Huntley J, Sommerlad A, et al Dementia prevention, intervention, and care: 2020 report of the Lancet Commission. Lancet. 2020;396(10248):413–446. 32738937 10.1016/S0140-6736(20)30367-6 PMC7392084

[14] Ritchie K, de Roquefeuil G, Ritchie C, et al COGNITO: Computerized assessment of information processing. J Psychol Psychother. 2014;4(2):1–5.

[15] Cole J . James-Cole/BrainageR: BrainageR v2.1. Zenodo; 2019. https://zenodo.org/record/3476365. Accessed 9 December 2022.

[16] Cole JH, Ritchie SJ, Bastin ME, et al Brain age predicts mortality. Mol Psychiatry. 2018;23(5):1385–1392. 28439103 10.1038/mp.2017.62 PMC5984097

[17] Niu X, Zhang F, Kounios J, Liang H. Improved prediction of brain age using multimodal neuroimaging data. Hum Brain Mapp. 2020;41(6):1626–1643. 31837193 10.1002/hbm.24899 PMC7267976

[18] Mak E, Dounavia ME, Operto G, et al APOE ɛ4 exacerbates age-dependent deficits in cortical microstructure. Brain Commun. 2024;6(1):fcad351. 38384997 10.1093/braincomms/fcad351 PMC10881196

[19] de Lange AMG, Cole JH. Commentary: Correction procedures in brain-age prediction. Neuroimage Clin. 2020;26:102229. 32120292 10.1016/j.nicl.2020.102229 PMC7049655

[20] Le TT, Kuplicki RT, McKinney BA, et al A nonlinear simulation framework supports adjusting for age when analyzing BrainAGE. Front Aging Neurosci. 2018;10:317. 30405393 10.3389/fnagi.2018.00317 PMC6208001

[21] de Lange AMG, Anatürk M, Rokicki J, et al Mind the gap: Performance metric evaluation in brain-age prediction. Hum Brain Mapp. 2022;43(10):3113–3129. 35312210 10.1002/hbm.25837 PMC9188975

[22] Klunk WE, Koeppe RA, Price JC, et al The centiloid project: Standardizing quantitative amyloid plaque estimation by PET. Alzheimers Dement. 2015;11(1):1–15.e1-4. 25443857 10.1016/j.jalz.2014.07.003 PMC4300247

[23] Fry A, Littlejohns TJ, Sudlow C, et al Comparison of sociodemographic and health-related characteristics of UK biobank participants with those of the general population. Am J Epidemiol. 2017;186(9):1026–1034. 28641372 10.1093/aje/kwx246 PMC5860371

[24] Dounavi ME, Newton C, Jenkins N, et al Macrostructural brain alterations at midlife are connected to cardiovascular and not inherited risk of future dementia: The PREVENT-dementia study. J Neurol. 2022;269(8):4299–4309. 35279756 10.1007/s00415-022-11061-7 PMC9294019

