# Supplementary material for: Brain age gap, dementia risk factors and cognition in middle age
Source: Brain Commun. 2024 Nov 19;6(6):fcae392. doi: 10.1093/braincomms/fcae392 (PMC11601159; doi:10.1093/braincomms/fcae392)
Supplement: fcae392_Supplementary_Data [file fcae392_supplementary_data.docx]

**Supplementary Methods**

**Missing data**

702 participants were recruited for the baseline dataset.

Number of participants with missing imaging=56 (n=646 remaining). Number of participants with incidental findings=11 (n=635 remaining). Number of participants with missing risk factor data: medical history of hypertension=2 (n=633 remaining); medical history of obesity=1 (n=632 remaining); medical history of hearing disorder=2 (n=630 remaining); *APOE4* carrier status=5 (n=625 remaining); alcohol intake=63 (n=562 remaining); head trauma=1 (n=561 remaining). Number of participants with missing cognitive data=9 (n=552 remaining). Thus, 552 participants were included.

**Multivariable predictive modelling**

Statistical associations do not necessarily imply predictive value. A range of machine learning (ML) algorithms were tested to investigate whether BAG and cognition could predict each other.

In the first set of analyses, ML algorithms were trained to predict BAG from the 17 raw COGNITO variables; algorithm performance was compared to a mean-predicting ‘Dummy’ baseline regressor to test the hypothesis that cognitive data can be used to predict BAG. In a second set of analyses, ML algorithms were trained to predict cognition PCs from demographic and risk-factor data (age, years of education, sex, scanning site, histories of hypertension, obesity and hearing disorder, *APOE4* carrier status, alcohol intake and head trauma); algorithm performance was compared to when BAG was additionally available, to test the hypothesis that BAG improves the prediction of cognitive performance when added to demographic/risk-factor data.

It is possible that any relationship between BAG and cognition is non-linear (rather than linear). Thus, in both sets of analyses, a range of linear (Ordinary Least Squares [OLS] Regression, Ridge Regression, Least Absolute Shrinkage and Selection Operator [LASSO] Regression) and non-linear (nu-Support Vector Regression [nuSVR], XGBoost Regression) ML algorithms were tested. The performance of linear and non-linear algorithms was compared to test the hypothesis that non-linear algorithms are superior to linear algorithms at explaining the relationship between BAG and cognition.

Random-permutation nested cross-validation (nested-CV) was used to provide an unbiased estimate of the mean generalisation error (defined as the Mean Absolute Deviation (MAD)) of each ‘algorithm-independent variables-target variable’ combination. Briefly, categorical variables were one-hot encoded. ‘ShuffleSplit’ produced five random train/test splits of the data (train_size=0.8) as five ‘outer-loop’ train/test datasets. For each ‘outer-loop’ train/test split, PCA (restricted to one output component) was performed on the training set COGNITO data to produce a training target PC, and the same PCA-parameters applied to the test set target data to produce a test target PC. Within each ‘outer-loop’ training dataset, ‘ShuffleSplit’ produced three random train/test splits of the data as three ‘inner-loop’ train/test datasets (train_size=0.66). The pipeline consisted of normalising independent variables to [0 1] (using ‘MinMaxScaler’) and then training the model to predict the target PC of the training dataset, before applying the same normalisation parameters to the test dataset and calculating the MAD of the trained model on the test target PC. Within the three ‘inner-loop’ train/test datasets of each ‘outer-loop’ train dataset, hyperparameters were optimised using ‘RandomizedSearchCV’ (20 random combinations of hyperparameter settings per ‘inner-loop’ train/test split).

95% Confidence Intervals (CIs) of each ‘algorithm-independent variables-target variable’ combination’s mean MAD were empirically estimated with bootstrapping (1000-iterations). Within each bootstrap iteration, the original dataset was bootstrapped to produce a ‘training dataset pool’ the same size as the original dataset; all samples not selected during bootstrapping for the training dataset were included in the ‘testing dataset pool’. From each ‘training dataset pool’, 5 ‘outer-loop’ training datasets were randomly sampled (with ‘ShuffleSplit’, train_size=0.8). From each ‘testing dataset pool’, 5 ‘outer-loop’ test datasets were randomly sampled (with ‘ShuffleSplit) to a size of 20% of the original dataset. Nested-CV occurred using these ‘outer loops’ as above. This process was repeated 1000 times to yield an empirical distribution of each ‘algorithm-independent variables-target variable’ combination’s mean MAD.

Both sets of prediction models tested the hypothesis that non-linear algorithms are superior to linear algorithms at explaining the relationship between BAG and cognition.

**Supplementary Results**

**Supplementary Table 1: Participant characteristics and unifactorial associations with Brain Age Gap**

| **Participant characteristic** | **Median (IQR) value or Number of participants** | **Median (IQR) Brain Age Gap** | **Association / Group Difference with Brain Age Gap** |
| --- | --- | --- | --- |
| **Brain Age** | 50.2 (9.8) | n/a | n/a |
| **Brain Age Gap** | -2.0 (7.8) | n/a | n/a |
| **Age** | 52.8 (8.7) | n/a | rho=-0.13, p=**0.003** |
| **Sex:**  Male  Female | 221  331 | -1.79 (9.33)  -2.11 (7.25) | p=0.91 |
| **Scanning site:**  Site A  Site B  Site C  Site D  Site E | 163  180  79  58  72 | -1.46 (7.72)  -0.90 (8.42)  -4.33 (9.61)  -2.96 (7.40)  -1.08 (6.83) | p=**0.0001**  post-hoc Dunn:  C<A (adj-p=0.006)  C<B (adj-p=7x10^-5^)  C<E (adj-p=0.005) |
| **Education** | 17 (5) | n/a | rho=-0.04, p=0.37 |
| **Medical History of Hypertension:**  No  Yes | 498  54 | -2.15 (7.93)  -0.29 (7.21) | p=0.02 |
| **Medical History of Obesity:**  No  Yes | 486  66 | -2.03 (8.47)  -1.94 (6.14) | p=0.54 |
| **Medical History of Hearing Disorder:**  No  Yes | 485  67 | -2.03 (7.94)  -1.80 (6.92) | p=0.81 |
| **Alcohol intake** | 4 (6) | n/a | rho=0.05, p=0.29 |
| **Head trauma** | 2 (4) | n/a | rho=-0.006, p=0.88 |
| ***APOE4* carrier status:**  Non-carrier  Carrier | 338  214 | -2.44 (8.34)  -1.09 (7.50) | p=0.04 |
| **Amyloid Centiloid Score (from n=95)** | 8.8 (13.9) | n/a | rho=0.07, p=0.48 |
|  |  |  |  |

Characteristics of the participants included in our analysis. Amyloid Centiloids, obtained from amyloid PET scans, was available for 95 of the 552 included participants. Scanning site names have been pseudonymised. Alcohol intake is the self-reported number of glasses of wine or beer consumed per week. Head trauma is the self-reported lifetime number of head blows. Age, Brain Age, Brain Age Gap and Education are provided in years. Association with Brain Age Gap was calculated as Spearman’s correlation for continuous variables. Group differences with Brain Age Gap were the results of Mann-Whitney U-test or Kruskall-Wallis H test for categorical variables with 2 or greater than 2 levels, respectively. Post-hoc Dunn test with Bonferroni correction was performed to identify which scanning sites had significantly different Brain Age Gap. Statistically significant p-values are highlighted in bold. The Bonferroni-corrected significance threshold was 0.004 (calculated as 0.05/11 since 11 unifactorial tests were performed). Abbreviations: *APOE4* = Apolipoprotein E epsilon 4 allele; BAG = Brain Age Gap; IQR = Interquartile Range; n = number of participants; n/a = not applicable; rho = Spearman’s rho.

**Supplementary Table 2: Post-hoc analysis - participant characteristics and unifactorial associations with ‘Age and Site Corrected Brain Age Gap’**

| **Participant characteristic** | **Median (IQR) value or Number of participants** | **Median (IQR)**  **‘Age and Site Corrected Brain Age Gap’** | **Association / Group Difference with ‘Age and Site Corrected Brain Age Gap’** |
| --- | --- | --- | --- |
| **‘Age and Site Corrected Brain Age’** | 52.1 (10.6) | n/a | n/a |
| **‘Age and Site Corrected**  **Brain Age Gap’** | -0.2 (8.1) | n/a | n/a |
| **Age** | 52.8 (8.7) | n/a | rho=-0.01, p=0.86 |
| **Sex:**  Male  Female | 221  331 | -0.11 (9.11)  -0.35 (7.43) | p=0.96 |
| **Race:** |  |  |  |
| Caucasian | 535 |  |  |
| Non-Caucasian | 17 |  |  |
| **Scanning site:**  Site A  Site B  Site C  Site D  Site E | 163  180  79  58  72 | -0.11 (8.08)  -0.38 (8.32)  -0.47 (8.73)  0.14 (7.99)  -0.10 (6.89) | p=0.99 |
| **Education** | 17 (5) | n/a | rho=-0.04, p=0.32 |
| **Medical History of Hypertension:**  No  Yes | 498  54 | -0.49 (7.88)  1.59 (7.75) | p=0.006 |
| **Medical History of Obesity:**  No  Yes | 486  66 | -0.18 (8.26)  -0.27 (6.87) | p=0.54 |
| **Medical History of Hearing Disorder:**  No  Yes | 485  67 | -0.20 (8.17)  -0.33 (7.51) | p=0.91 |
| **Alcohol intake** | 4 (6) | n/a | rho=0.05, p=0.21 |
| **Head trauma** | 2 (4) | n/a | rho=-0.03, p=0.46 |
| ***APOE4* carrier status:**  Non-carrier  Carrier | 338  214 | -0.50 (7.80)  0.21 (7.98) | p=0.12 |
| ***APOE4* homozygous status:** |  |  | p=0.69 |
| Non-homozygous | 525 | -0.20 (8.12) |  |
| Homozygous | 27 | 0.26 (7.06) |  |
| **Amyloid Centiloid Score (from n=95)** | 8.8 (13.9) | n/a | rho=-0.02, p=0.88 |
|  |  |  |  |

Characteristics of the participants included in our analysis. Amyloid Centiloids, obtained from amyloid PET scans, was available for 95 of the 552 included participants. Scanning site names have been pseudonymised. Alcohol intake is the self-reported number of glasses of wine or beer consumed per week. Head trauma is the self-reported lifetime number of head blows. Age, ‘Age and Site Corrected Brain Age’, ‘Age and Site Corrected Brain Age Gap’ and Education are provided in years. Association with ‘Age and Site Corrected Brain Age Gap’ was calculated as Spearman’s correlation for continuous variables. Group differences with ‘Age and Site Corrected Brain Age Gap’ were the results of Mann-Whitney U-test or Kruskall-Wallis H test for categorical variables with 2 or greater than 2 levels, respectively. Statistically significant p-values are highlighted in bold. The Bonferroni-corrected significance threshold was 0.004 (calculated as 0.05/12 since 13 unifactorial tests were performed in this post-hoc analysis). Abbreviations: *APOE4* = Apolipoprotein E epsilon 4; BAG = Brain Age Gap; IQR = Interquartile Range; n = number of participants; n/a = not applicable; rho = Spearman’s rho.

**Supplementary Table 3: Post-hoc analysis - Multiple Linear Regression model of participant characteristics and modifiable dementia risk-factors with ‘Age and Site Corrected Brain Age Gap’**

| **Participant characteristic** | **Multiple linear regression with ‘Age and Site Corrected Brain Age Gap’** | |
| --- | --- | --- |
|  | **B (95% CI)** | **P value** |
| **Intercept** | 0.34 (-2.45 to 3.13) | 0.81 |
| **Medical History of Hypertension** | 2.23 (0.54 to 3.91) | 0.01 |
| **Medical History of Obesity** | 0.21 (-1.34 to 1.75) | 0.79 |
| **Medical History of Hearing Disorder** | 0.22 (-1.30 to 1.74) | 0.78 |
| **Sex - Male** | -0.37 (-1.43 to 0.68) | 0.49 |
| **Alcohol** | 0.10 (0.02 to 0.18) | 0.01 |
| **Head trauma** | 0.02 (-0.09 to 0.12) | 0.76 |
| **Years of Education** | -0.08 (-0.23 to 0.08) | 0.34 |

Robust multiple linear regression of participant characteristics and modifiable dementia risk factors against ‘Age and Site Corrected Brain Age Gap’. This analysis was performed on the 552 included participants. Statistically significant p-values are highlighted in bold. Abbreviations: B = unstandardised regression coefficient; CI = confidence interval.

**Supplementary Table 4: Multiple Linear Regression model of participant characteristics and *APOE4* with Brain Age Gap**

| **Participant characteristic** | **Multiple linear regression with Brain Age Gap** | |
| --- | --- | --- |
|  | **B (95% CI)** | **P value** |
| **Intercept** | -18.34 (-63.49 to 26.80) | 0.43 |
| ***APOE4* carrier** | 0.79 (-0.25 to 1.82) | 0.14 |
| **Sex - Male** | -4.11 (-13.90 to 5.68) | 0.41 |
| **Scanning Site (compared to site A):**  Site B  Site C  Site D  Site E | 0.93 (-0.36 to 2.21)  -2.85 (-4.49 to -1.22)  -1.28 (-3.08 to 0.52)  0.46 (-1.25 to 2.18) | 0.16  **0.001**  0.16  0.60 |
| **Age** | 0.97 (-0.83 to 2.76) | 0.29 |
| **Age*Sex** | -0.08 (-0.27 to 0.11) | 0.40 |
| **Years of Education** | -0.08 (-0.24 to 0.09) | 0.35 |
| **Age^2^** | -0.01 (-0.03 to 0.01) | 0.24 |

Robust multiple linear regression of participant characteristics and *APOE4* against Brain Age Gap. This analysis was performed on the 552 included participants. Statistically significant p-values are highlighted in bold. Abbreviations: *APOE4* = Apolipoprotein E epsilon 4; B = unstandardised regression coefficient; CI = confidence interval.

**Supplementary Table 5: Post-hoc analysis - Multiple Linear Regression model of participant characteristics and *APOE4* with ‘Age and Site Corrected Brain Age Gap’**

| **Participant characteristic** | **Multiple linear regression with ‘Age and Site Corrected Brain Age Gap’** | |
| --- | --- | --- |
|  | **B (95% CI)** | **P value** |
| **Intercept** | 1.23 (-1.49 to 3.94) | 0.38 |
| ***APOE4* homozygous** | 0.48 (-1.83 to 2.80) | 0.68 |
| **Sex - Male** | 0.02 (-1.00 to 1.04) | 0.97 |
| **Years of Education** | -0.08 (-0.24 to 0.07) | 0.29 |

Robust multiple linear regression of participant characteristics and *APOE4* homozygosity against ‘Age and Site Corrected Brain Age Gap’. This analysis was performed on the 552 included participants. Statistically significant p-values are highlighted in bold. Abbreviations: *APOE4* = Apolipoprotein E epsilon 4; B = unstandardised regression coefficient; CI = confidence interval.

**Supplementary Table 6: Multiple Linear Regression model of participant characteristics and Amyloid Centiloid Score with Brain Age Gap**

| **Participant characteristic** | **Multiple linear regression with Brain Age Gap** | |
| --- | --- | --- |
|  | **B (95% CI)** | **P value** |
| **Intercept** | 33.44 (-109.73 to 176.61) | 0.65 |
| **Sex - Male** | -25.42 (-61.68 to 10.83) | 0.17 |
| **Scanning Site (compared to site A):**  Site B  Site C  Site D  Site E | -0.37 (-6.91 to 6.16)  -3.69 (-6.58 to -0.80)  0.73 (-3.76 to 5.23)  - | 0.91  0.01  0.75  - |
| **Amyloid Centiloid Score** | -0.03 (-0.11 to 0.04) | 0.39 |
| **Age** | -0.31 (-6.26 to 5.64) | 0.92 |
| **Age*Sex** | -0.45 (-1.13 to 0.22) | 0.19 |
| **Years of Education** | -0.11 (-0.56 to 0.34) | 0.63 |
| **Age^2^** | 0.003 (-0.05 to 0.06) | 0.92 |

Robust multiple linear regression of participant characteristics and Amyloid Centiloids against Brain Age Gap. This analysis was performed on the 95 included participants for whom Amyloid Centiloids were available. Site E had no participants with Amyloid Centilod values. Statistically significant p-values are highlighted in bold. Abbreviations: B = unstandardised regression coefficient; CI = confidence interval.

**Supplementary Table 7: Post-hoc analysis - Multiple Linear Regression model of participant characteristics and Amyloid Centiloid Score with ‘Age and Site Corrected Brain Age Gap’**

| **Participant characteristic** | **Multiple linear regression with ‘Age and Site Corrected Brain Age Gap’** | |
| --- | --- | --- |
|  | **B (95% CI)** | **P value** |
| **Intercept** | 4.75 (-2.28 to 11.79) | 0.19 |
| **Sex - Male** | -1.44 (-3.88 to 1.00) | 0.25 |
| **Amyloid Centiloid Score** | -0.04 (-0.11 to 0.03) | 0.32 |
| **Years of Education** | -0.22 (-0.63 to 0.19) | 0.30 |

Robust multiple linear regression of participant characteristics and Amyloid Centiloids against ‘Age and Site Corrected Brain Age Gap’. This analysis was performed on the 95 included participants for whom Amyloid Centiloids were available. Statistically significant p-values are highlighted in bold. Abbreviations: B = unstandardised regression coefficient; CI = confidence interval.

**Supplementary Table 8: Principal Component Loadings of COGNITO variables**

| **COGNITO variable** | **Cognition PC** | **Attention PC** | **Language PC** | **Memory PC** | **Visuospatial PC** |
| --- | --- | --- | --- | --- | --- |
| **Attention:** |  |  |  |  |  |
| **Visual** | -0.03 | -0.50 |  |  |  |
| **Auditory** | -0.14 | -0.48 |  |  |  |
| **Visual and Auditory** | -0.06 | -0.72 |  |  |  |
| **Language:** |  |  |  |  |  |
| **Sentence Comprehension** | -0.16 |  | -0.51 |  |  |
| **Verbal Fluency** | -0.30 |  | -0.58 |  |  |
| **Vocabulary Test** | -0.30 |  | -0.63 |  |  |
| **Memory:** |  |  |  |  |  |
| **Immediate Recall** | -0.42 |  |  | -0.53 |  |
| **Delayed Recall** | -0.41 |  |  | -0.52 |  |
| **Face Recall** | -0.23 |  |  | -0.29 |  |
| **Name-Face Associations** | -0.40 |  |  | -0.51 |  |
| **Narrative Recall** | -0.33 |  |  | -0.33 |  |
| **Implicit Memory** | 0.01 |  |  | -0.02 |  |
| **Visuospatial ability** |  |  |  |  |  |
| **Form Matching** | -0.13 |  |  |  | -0.63 |
| **Span** | -0.07 |  |  |  | -0.35 |
| **Logical Series** | -0.27 |  |  |  | -0.64 |
| **Construction** | -0.02 |  |  |  | -0.04 |
| **Stroop Test** | 0.10 |  |  |  | 0.27 |
|  |  |  |  |  |  |
| **Proportion of variance explained (%)** | 16.4 | 47.7 | 47.2 | 38.2 | 26.0 |
| **Eigenvalue** | 2.79 | 1.43 | 1.42 | 2.29 | 1.30 |
|  |  |  |  |  |  |

Principal Component Loading Scores of COGNITO variables resulting from unrotated Principal Component Analysis (restricted to 1 component) of all 17 variables (‘Cognition PC’) and attention (‘Attention PC’), language (‘Language PC’), memory (‘Memory PC’) and visuospatial (‘Visuospatial PC’) subsets. This analysis was performed on the 552 included participants. The proportion of variance explained, and eigenvalue, of each PC are provided at the bottom. Abbreviations: PC = Principal Component.

**Supplementary Table 9: Multiple Linear Regression model of participant characteristics and Brain Age Gap with ‘Attention PC’**

| **Participant characteristic** | **Multiple linear regression with ‘Attention PC’** | |
| --- | --- | --- |
|  | **B (95% CI)** | **P value** |
| **Intercept** | -2.33 (-6.37 to 1.72) | 0.26 |
| **Sex - Male** | -0.10 (-0.97 to 0.78) | 0.83 |
| **Scanning Site (compared to site A):**  Site B  Site C  Site D  Site E | -0.07 (-0.19 to 0.05)  -0.10 (-0.25 to 0.05)  -0.04 (-0.21 to 0.12)  -0.08 (-0.24 to 0.07) | 0.23  0.17  0.59  0.28 |
| **Brain Age Gap** | 0.005 (-0.003 to 0.01) | 0.25 |
| **Age** | 0.08 (-0.08 to 0.24) | 0.32 |
| **Age*Sex** | 0.001 (-0.02 to 0.02) | 0.92 |
| **Years of Education** | -0.007 (-0.02 to 0.008) | 0.38 |
| **Age^2^** | -0.001 (-0.002 to 0.001) | 0.38 |

Robust multiple linear regression of participant characteristics and Brain Age Gap against ‘Attention PC’. This analysis was performed on the 552 included participants. Statistically significant p-values are highlighted in bold. Abbreviations: B = unstandardised regression coefficient; CI = confidence interval; PC = Principal Component.

**Supplementary Table 10: Multiple Linear Regression model of participant characteristics and Brain Age Gap with ‘Language PC’**

| **Participant characteristic** | **Multiple linear regression with ‘Language PC’** | |
| --- | --- | --- |
|  | **B (95% CI)** | **P value** |
| **Intercept** | 7.75 (-0.03 to 15.53) | 0.05 |
| **Sex - Male** | -1.36 (-3.05 to 0.33) | 0.12 |
| **Scanning Site (compared to site A):**  Site B  Site C  Site D  Site E | 0.26 (0.03 to 0.48)  -0.003 (-0.29 to 0.28)  -0.06 (-0.37 to 0.36)  0.69 (0.40 to 0.99) | **0.02**  0.98  0.73  **4.1x10^-6^** |
| **Brain Age Gap** | 0.01 (-0.002 to 0.03) | 0.09 |
| **Age** | -0.16 (-0.47 to 0.15) | 0.32 |
| **Age*Sex** | -0.03 (-0.06 to 0.01) | 0.09 |
| **Years of Education** | -0.13 (-0.16 to -0.10) | **1.4x10^-20^** |
| **Age^2^** | 0.001 (-0.002 to 0.004) | 0.36 |

Robust multiple linear regression of participant characteristics and Brain Age Gap against ‘Language PC’. This analysis was performed on the 552 included participants. Statistically significant p-values are highlighted in bold. Abbreviations: B = unstandardised regression coefficient; CI = confidence interval; PC = Principal Component.

**Supplementary Table 11: Multiple Linear Regression model of participant characteristics and Brain Age Gap with ‘Memory PC’**

| **Participant characteristic** | **Multiple linear regression with ‘Memory PC’** | |
| --- | --- | --- |
|  | **B (95% CI)** | **P value** |
| **Intercept** | 6.46 (-4.85 to 17.76) | 0.26 |
| **Sex - Male** | -0.21 (-2.67 to 2.24) | 0.86 |
| **Scanning Site (compared to site A):**  Site B  Site C  Site D  Site E | -0.38 (-0.71 to -0.06)  0.31 (-0.11 to 0.72)  -0.09 (-0.54 to 0.37)  0.27 (-0.16 to 0.70) | **0.02**  0.15  0.71  0.22 |
| **Brain Age Gap** | -0.01 (-0.03 to 0.02) | 0.61 |
| **Age** | -0.24 (-0.68 to 0.21) | 0.30 |
| **Age*Sex** | -0.02 (-0.07 to 0.03) | 0.40 |
| **Years of Education** | -0.06 (-0.10 to -0.02) | **0.003** |
| **Age^2^** | 0.003 (-0.002 to 0.007) | 0.22 |

Robust multiple linear regression of participant characteristics and Brain Age Gap against ‘Memory PC’. This analysis was performed on the 552 included participants. Statistically significant p-values are highlighted in bold. Abbreviations: B = unstandardised regression coefficient; CI = confidence interval; PC = Principal Component.**Supplementary Table 12: Multiple Linear Regression model of participant characteristics and Brain Age Gap with ‘Visuospatial PC’**

| **Participant characteristic** | **Multiple linear regression with ‘Visuospatial PC’** | |
| --- | --- | --- |
|  | **B (95% CI)** | **P value** |
| **Intercept** | 4.50 (-4.01 to 13.00) | 0.30 |
| **Sex - Male** | -0.23 (-2.08 to 1.62) | 0.81 |
| **Scanning Site (compared to site A):**  Site B  Site C  Site D  Site E | -0.03 (-0.27 to 0.22)  0.10 (-0.21 to 0.41)  -0.03 (-0.37 to 0.31)  0.26 (-0.06 to 0.59) | 0.83  0.53  0.87  0.11 |
| **Brain Age Gap** | 0.01 (-0.01 to 0.03) | 0.18 |
| **Age** | -0.16 (-0.50 to 0.18) | 0.35 |
| **Age*Sex** | 5x10^-4^ (-0.04 to 0.04) | 0.98 |
| **Years of Education** | -0.07 (-0.10 to -0.04) | **1.2x10^-5^** |
| **Age^2^** | 0.002 (-0.001 to 0.005) | 0.27 |

Robust multiple linear regression of participant characteristics and Brain Age Gap against ‘Visuospatial PC’. This analysis was performed on the 552 included participants. Statistically significant p-values are highlighted in bold. Abbreviations: B = unstandardised regression coefficient; CI = confidence interval; PC = Principal Component.

**Supplementary Table 13: Post-hoc analysis - Multiple Linear Regression model of participant characteristics and ‘Age and Site Corrected Brain Age Gap’ with ‘Cognition PC’**

| **Participant characteristic** | **Multiple linear regression with ‘Cognition PC’** | |
| --- | --- | --- |
|  | **B (95% CI)** | **P value** |
| **Intercept** | 11.92 (-0.03 to 23.88) | 0.05 |
| **Sex - Male** | -0.84 (-3.43 to 1.76) | 0.53 |
| **Scanning Site (compared to site A):**  Site B  Site C  Site D  Site E | -0.20 (-0.54 to 0.15)  0.36 (-0.08 to 0.79)  -0.03 (-0.51 to 0.44)  0.69 (0.24 to 1.14) | 0.26  0.11  0.89  **0.003** |
| **‘Age and Site Corrected Brain Age Gap’** | 0.004 (-0.02 to 0.03) | 0.72 |
| **Age** | -0.37 (-0.85 to 0.10) | 0.12 |
| **Age*Sex** | -0.03 (-0.08 to 0.02) | 0.26 |
| **Years of Education** | -0.14 (-0.19 to -0.10) | **4.5x10^-11^** |
| **Age^2^** | 0.004 (-0.001 to 0.009) | 0.09 |

Robust multiple linear regression of participant characteristics and ‘Age and Site Corrected Brain Age Gap’ against ‘Cognition PC’. This analysis was performed on the 552 included participants. Statistically significant p-values are highlighted in bold. Abbreviations: B = unstandardised regression coefficient; CI = confidence interval; PC = Principal Component.

**Supplementary Table 14: Post-hoc analysis - Multiple Linear Regression model of participant characteristics and ‘Age and Site Corrected Brain Age Gap’ with ‘Attention PC’**

| **Participant characteristic** | **Multiple linear regression with ‘Attention PC’** | |
| --- | --- | --- |
|  | **B (95% CI)** | **P value** |
| **Intercept** | -2.30 (-6.35 to 1.74) | 0.26 |
| **Sex - Male** | -0.10 (-0.98 to 0.78) | 0.83 |
| **Scanning Site (compared to site A):**  Site B  Site C  Site D  Site E | -0.07 (-0.18 to 0.05)  -0.12 (-0.26 to 0.03)  -0.05 (-0.21 to 0.11)  -0.08 (-0.24 to 0.07) | 0.26  0.12  0.54  0.29 |
| **‘Age and Site Corrected Brain Age Gap’** | 0.004 (-0.003 to 0.01) | 0.28 |
| **Age** | 0.08 (-0.08 to 0.24) | 0.32 |
| **Age*Sex** | 0.0008 (-0.02 to 0.02) | 0.93 |
| **Years of Education** | -0.007 (-0.02 to 0.008) | 0.37 |
| **Age^2^** | -0.0007 (-0.002 to 0.001) | 0.38 |

Robust multiple linear regression of participant characteristics and ‘Age and Site Corrected Brain Age Gap’ against ‘Attention PC’. This analysis was performed on the 552 included participants. Statistically significant p-values are highlighted in bold. Abbreviations: B = unstandardised regression coefficient; CI = confidence interval; PC = Principal Component.

**Supplementary Table 15: Post-hoc analysis - Multiple Linear Regression model of participant characteristics and ‘Age and Site Corrected Brain Age Gap’ with ‘Language PC’**

| **Participant characteristic** | **Multiple linear regression with ‘Language PC’** | |
| --- | --- | --- |
|  | **B (95% CI)** | **P value** |
| **Intercept** | 7.80 (0.01 to 15.60) | 0.05 |
| **Sex - Male** | -1.36 (-3.05 to 0.34) | 0.12 |
| **Scanning Site (compared to site A):**  Site B  Site C  Site D  Site E | 0.27 (0.04 to 0.49)  -0.04 (-0.32 to 0.24)  -0.07 (-0.38 to 0.24)  0.70 (0.40 to 1.00) | **0.02**  0.79  0.65  **3.6x10^-6^** |
| **‘Age and Site Corrected Brain Age Gap’** | 0.01 (-0.002 to 0.03) | 0.09 |
| **Age** | -0.16 (-0.47 to 0.15) | 0.31 |
| **Age*Sex** | -0.03 (-0.06 to 0.01) | 0.09 |
| **Years of Education** | -0.13 (-0.16 to -0.10) | **1.4x10^-20^** |
| **Age^2^** | 0.001 (-0.002 to 0.004) | 0.36 |

Robust multiple linear regression of participant characteristics and ‘Age and Site Corrected Brain Age Gap’ against ‘Language PC’. This analysis was performed on the 552 included participants. Statistically significant p-values are highlighted in bold. Abbreviations: B = unstandardised regression coefficient; CI = confidence interval; PC = Principal Component.

**Supplementary Table 16: Post-hoc analysis - Multiple Linear Regression model of participant characteristics and ‘Age and Site Corrected Brain Age Gap’ with ‘Memory PC’**

| **Participant characteristic** | **Multiple linear regression with ‘Memory PC’** | |
| --- | --- | --- |
|  | **B (95% CI)** | **P value** |
| **Intercept** | 6.45 (-4.85 to 17.75) | 0.26 |
| **Sex - Male** | -0.21 (-2.66 to 2.24) | 0.87 |
| **Scanning Site (compared to site A):**  Site B  Site C  Site D  Site E | -0.39 (-0.71 to -0.07)  0.32 (-0.09 to 0.73)  -0.08 (-0.53 to 0.37)  0.27 (-0.16 to 0.70) | **0.02**  0.12  0.73  0.22 |
| **‘Age and Site Corrected Brain Age Gap’** | -0.01 (-0.03 to 0.02) | 0.65 |
| **Age** | -0.24 (-0.68 to 0.21) | 0.30 |
| **Age*Sex** | -0.02 (-0.07 to 0.03) | 0.40 |
| **Years of Education** | -0.06 (-0.10 to -0.02) | **0.003** |
| **Age^2^** | 0.003 (-0.002 to 0.01) | 0.22 |

Robust multiple linear regression of participant characteristics and ‘Age and Site Corrected Brain Age Gap’ against ‘Memory PC’. This analysis was performed on the 552 included participants. Statistically significant p-values are highlighted in bold. Abbreviations: B = unstandardised regression coefficient; CI = confidence interval; PC = Principal Component.

**Supplementary Table 17: Post-hoc analysis - Multiple Linear Regression model of participant characteristics and ‘Age and Site Corrected Brain Age Gap’ with ‘Visuospatial PC’**

| **Participant characteristic** | **Multiple linear regression with ‘Visuospatial PC’** | |
| --- | --- | --- |
|  | **B (95% CI)** | **P value** |
| **Intercept** | 4.57 (-3.96 to 13.10) | 0.29 |
| **Sex - Male** | -0.24 (-2.09 to 1.61) | 0.80 |
| **Scanning Site (compared to site A):**  Site B  Site C  Site D  Site E | -0.02 (-0.26 to 0.23)  0.07 (-0.24 to 0.38)  -0.04 (-0.39 to 0.30)  0.27 (-0.06 to 0.59) | 0.89  0.66  0.80  0.10 |
| **‘Age and Site Corrected Brain Age Gap’** | 0.01 (-0.01 to 0.03) | 0.20 |
| **Age** | -0.16 (-0.50 to 0.18) | 0.35 |
| **Age*Sex** | -0.0008 (-0.04 to 0.04) | 0.97 |
| **Years of Education** | -0.07 (-0.10 to -0.04) | **1.2x10^-5^** |
| **Age^2^** | 0.001 (-0.001 to 0.005) | 0.27 |

Robust multiple linear regression of participant characteristics and ‘Age and Site Corrected Brain Age Gap’ against ‘Visuospatial PC’. This analysis was performed on the 552 included participants. Statistically significant p-values are highlighted in bold. Abbreviations: B = unstandardised regression coefficient; CI = confidence interval; PC = Principal Component.

**Supplementary Table 18: Multivariable regression models predicting ‘Cognition PC’ from demographic, risk-factor and Brain Age Gap data**

| **Dependent variable** | **Independent variables** | **Regression Algorithm** | **Mean MAD** | **95% CI of**  **Mean MAD** |
| --- | --- | --- | --- | --- |
|  |  |  |  |  |
| Cognition PC | Demographic/risk-factor | OLS | 1.25 | 1.14 to 1.42 |
| Cognition PC | Demographic/risk-factor + BAG | OLS | 1.28 | 1.14 to 1.42 |
| Cognition PC | Demographic/risk-factor | Ridge | 1.32 | 1.14 to 1.41 |
| Cognition PC | Demographic/risk-factor + BAG | Ridge | 1.32 | 1.14 to 1.41 |
| Cognition PC | Demographic/risk-factor | LASSO | 1.25 | 1.14 to 1.41 |
| Cognition PC | Demographic/risk-factor + BAG | LASSO | 1.25 | 1.14 to 1.41 |
| Cognition PC | Demographic/risk-factor | NuSVR | 1.33 | 1.24 to 1.54 |
| Cognition PC | Demographic/risk-factor + BAG | NuSVR | 1.24 | 1.25 to 1.55 |
| Cognition PC | Demographic/risk-factor | XGBoost | 1.28 | 1.32 to 1.62 |
| Cognition PC | Demographic/risk-factor + BAG | XGBoost | 1.23 | 1.31 to 1.59 |
|  |  |  |  |  |

Multivariable regression models predicting ‘Cognition PC’ from demographic, risk-factor and Brain Age Gap data. Demographic and risk-factor variables were: age; years of education; sex; scanning site; history of hypertension; history of obesity; history of hearing disorder; *APOE4* carrier status; alcohol intake; and head trauma. This analysis was performed on the 552 included participants. Abbreviations: BAG = Brain Age Gap; CI = confidence interval; LASSO = Least Absolute Shrinkage and Selection Operator; MAD = Mean Absolute Deviation; NuSVR = nu-Support Vector Regression; OLS = Ordinary Least Squares; PC = Principal Component; XGBoost = Extreme Gradient Boosting.

**Supplementary Table 19: Multivariable regression models predicting ‘Attention PC’ from demographic, risk-factor and Brain Age Gap data**

| **Dependent variable** | **Independent variables** | **Regression Algorithm** | **Mean MAD** | **95% CI of**  **Mean MAD** |
| --- | --- | --- | --- | --- |
|  |  |  |  |  |
| Attention PC | Demographic/risk-factor | OLS | 0.66 | 0.51 to 1.07 |
| Attention PC | Demographic/risk-factor + BAG | OLS | 0.59 | 0.52 to 1.07 |
| Attention PC | Demographic/risk-factor | Ridge | 0.61 | 0.50 to 1.06 |
| Attention PC | Demographic/risk-factor + BAG | Ridge | 0.63 | 0.51 to 1.06 |
| Attention PC | Demographic/risk-factor | LASSO | 0.59 | 0.49 to 1.06 |
| Attention PC | Demographic/risk-factor + BAG | LASSO | 0.64 | 0.49 to 1.06 |
| Attention PC | Demographic/risk-factor | NuSVR | 0.67 | 0.36 to 0.92 |
| Attention PC | Demographic/risk-factor + BAG | NuSVR | 0.49 | 0.36 to 0.93 |
| Attention PC | Demographic/risk-factor | XGBoost | 0.60 | 0.57 to 1.14 |
| Attention PC | Demographic/risk-factor + BAG | XGBoost | 0.60 | 0.54 to 1.13 |
|  |  |  |  |  |

Multivariable regression models predicting ‘Attention PC’ from demographic, risk-factor and Brain Age Gap data. Demographic and risk-factor variables were: age; years of education; sex; scanning site; history of hypertension; history of obesity; history of hearing disorder; *APOE4* carrier status; alcohol intake; and head trauma. This analysis was performed on the 552 included participants. Abbreviations: BAG = Brain Age Gap; CI = confidence interval; LASSO = Least Absolute Shrinkage and Selection Operator; MAD = Mean Absolute Deviation; NuSVR = nu-Support Vector Regression; OLS = Ordinary Least Squares; PC = Principal Component; XGBoost = Extreme Gradient Boosting.

**Supplementary Table 20: Multivariable regression models predicting ‘Language PC’ from demographic, risk-factor and Brain Age Gap data**

| **Dependent variable** | **Independent variables** | **Regression Algorithm** | **Mean MAD** | **95% CI of**  **Mean MAD** |
| --- | --- | --- | --- | --- |
|  |  |  |  |  |
| Language PC | Demographic/risk-factor | OLS | 0.88 | 0.76 to 0.96 |
| Language PC | Demographic/risk-factor + BAG | OLS | 0.85 | 0.76 to 0.96 |
| Language PC | Demographic/risk-factor | Ridge | 0.85 | 0.75 to 0.96 |
| Language PC | Demographic/risk-factor + BAG | Ridge | 0.85 | 0.76 to 0.96 |
| Language PC | Demographic/risk-factor | LASSO | 0.85 | 0.75 to 0.96 |
| Language PC | Demographic/risk-factor + BAG | LASSO | 0.80 | 0.76 to 0.97 |
| Language PC | Demographic/risk-factor | NuSVR | 0.82 | 0.82 to 1.06 |
| Language PC | Demographic/risk-factor + BAG | NuSVR | 0.86 | 0.83 to 1.07 |
| Language PC | Demographic/risk-factor | XGBoost | 0.90 | 0.90 to 1.11 |
| Language PC | Demographic/risk-factor + BAG | XGBoost | 0.84 | 0.87 to 1.08 |
|  |  |  |  |  |

Multivariable regression models predicting ‘Language PC’ from demographic, risk-factor and Brain Age Gap data. Demographic and risk-factor variables were: age; years of education; sex; scanning site; history of hypertension; history of obesity; history of hearing disorder; *APOE4* carrier status; alcohol intake; and head trauma. This analysis was performed on the 552 included participants. Abbreviations: BAG = Brain Age Gap; CI = confidence interval; LASSO = Least Absolute Shrinkage and Selection Operator; MAD = Mean Absolute Deviation; NuSVR = nu-Support Vector Regression; OLS = Ordinary Least Squares; PC = Principal Component; XGBoost = Extreme Gradient Boosting.

**Supplementary Table 21: Multivariable regression models predicting ‘Memory PC’ from demographic, risk-factor and Brain Age Gap data**

| **Dependent variable** | **Independent variables** | **Regression Algorithm** | **Mean MAD** | **95% CI of**  **Mean MAD** |
| --- | --- | --- | --- | --- |
|  |  |  |  |  |
| Memory PC | Demographic/risk-factor | OLS | 1.14 | 1.02 to 1.30 |
| Memory PC | Demographic/risk-factor + BAG | OLS | 1.12 | 1.02 to 1.30 |
| Memory PC | Demographic/risk-factor | Ridge | 1.11 | 1.02 to 1.30 |
| Memory PC | Demographic/risk-factor + BAG | Ridge | 1.14 | 1.02 to 1.30 |
| Memory PC | Demographic/risk-factor | LASSO | 1.07 | 1.02 to 1.29 |
| Memory PC | Demographic/risk-factor + BAG | LASSO | 1.19 | 1.02 to 1.30 |
| Memory PC | Demographic/risk-factor | NuSVR | 1.11 | 1.12 to 1.40 |
| Memory PC | Demographic/risk-factor + BAG | NuSVR | 1.17 | 1.12 to 1.42 |
| Memory PC | Demographic/risk-factor | XGBoost | 1.20 | 1.22 to 1.51 |
| Memory PC | Demographic/risk-factor + BAG | XGBoost | 1.16 | 1.21 to 1.48 |
|  |  |  |  |  |

Multivariable regression models predicting ‘Memory PC’ from demographic, risk-factor and Brain Age Gap data. Demographic and risk-factor variables were: age; years of education; sex; scanning site; history of hypertension; history of obesity; history of hearing disorder; *APOE4* carrier status; alcohol intake; and head trauma. This analysis was performed on the 552 included participants. Abbreviations: BAG = Brain Age Gap; CI = confidence interval; LASSO = Least Absolute Shrinkage and Selection Operator; MAD = Mean Absolute Deviation; NuSVR = nu-Support Vector Regression; OLS = Ordinary Least Squares; PC = Principal Component; XGBoost = Extreme Gradient Boosting.

**Supplementary Table 22: Multivariable regression models predicting ‘Visuospatial PC’ from demographic, risk-factor and Brain Age Gap data**

| **Dependent variable** | **Independent variables** | **Regression Algorithm** | **Mean MAD** | **95% CI of**  **Mean MAD** |
| --- | --- | --- | --- | --- |
|  |  |  |  |  |
| Visuospatial PC | Demographic/risk-factor | OLS | 0.91 | 0.78 to 1.00 |
| Visuospatial PC | Demographic/risk-factor + BAG | OLS | 0.86 | 0.79 to 1.00 |
| Visuospatial PC | Demographic/risk-factor | Ridge | 0.85 | 0.78 to 1.00 |
| Visuospatial PC | Demographic/risk-factor + BAG | Ridge | 0.91 | 0.78 to 0.99 |
| Visuospatial PC | Demographic/risk-factor | LASSO | 0.87 | 0.78 to 0.99 |
| Visuospatial PC | Demographic/risk-factor + BAG | LASSO | 0.90 | 0.78 to 0.99 |
| Visuospatial PC | Demographic/risk-factor | NuSVR | 0.88 | 0.85 to 1.07 |
| Visuospatial PC | Demographic/risk-factor + BAG | NuSVR | 0.88 | 0.85 to 1.07 |
| Visuospatial PC | Demographic/risk-factor | XGBoost | 0.90 | 0.91 to 1.14 |
| Visuospatial PC | Demographic/risk-factor + BAG | XGBoost | 0.89 | 0.90 to 1.11 |
|  |  |  |  |  |

Multivariable regression models predicting ‘Visuospatial PC’ from demographic, risk-factor and Brain Age Gap data. Demographic and risk-factor variables were: age; years of education; sex; scanning site; history of hypertension; history of obesity; history of hearing disorder; *APOE4* carrier status; alcohol intake; and head trauma. This analysis was performed on the 552 included participants. Abbreviations: BAG = Brain Age Gap; CI = confidence interval; LASSO = Least Absolute Shrinkage and Selection Operator; MAD = Mean Absolute Deviation; NuSVR = nu-Support Vector Regression; OLS = Ordinary Least Squares; PC = Principal Component; XGBoost = Extreme Gradient Boosting.
